# Recognition of the dense fine speckled (DFS) pattern remains challenging: results from an international internet-based survey

**DOI:** 10.1007/s13317-016-0081-2

**Published:** 2016-07-09

**Authors:** Chelsea Bentow, Marvin J. Fritzler, Eckart Mummert, Michael Mahler

**Affiliations:** 1 Department of Research and Development, Inova Diagnostics, 9900 Old Grove Road, San Diego, CA 92131-1638 USA; 2 Cumming School of Medicine, University of Calgary, Calgary, T2N 4N1 Canada

**Keywords:** Antinuclear antibodies, DFS, LEDGF, Autoantibodies, Autoimmune disease, Indirect immunofluorescence

## Abstract

**Purpose:**

The dense fine speckled (DFS) pattern as detected by indirect immunofluorescence (IIF) on HEp-2 cells has been associated with several inflammatory diseases but is most commonly observed in individuals that do not have an antinuclear antibody (ANA)-associated rheumatic disease and even in apparently healthy individuals. Consequently, the accurate identification and correct reporting of this IIF pattern is of utmost importance and accordingly has been recognized by several international study groups for the detection of ANA. Furthermore, the DFS IIF pattern has recently been recommended as a competency level recognition pattern by the International Consensus on Antinuclear Antibody (ANA) Pattern (ICAP, http://www.anapatterns.org/) Committee. The objective of this study was to use an internet-based survey to assess how accurately the DFS IIF pattern was recognized by experienced technologists.

**Methods:**

High-resolution digital IIF images were captured using the automated IIF NOVA View instrument (Inova Diagnostics, San Diego, CA). Ten images were posted in an anonymous, international, internet-based interpretive survey. Two hundred and thirty IIF technologists were invited to participate. Four of the images in the survey were from previously characterized serum samples with classical ANA IIF patterns (nucleolar, centromere, homogeneous, and speckled) and two of the images were from samples with a DFS IIF ANA pattern and isolated anti-DFS70 antibodies as determined by a chemiluminescence immunoassay. The remaining four images were from sera with the classic IIF ANA patterns referred to above and mixed with a monospecific anti-DFS70-positive sample. The survey included multiple choice selections: homogeneous, DFS, centromere, nucleolar, speckled, other, or unrecognizable.

**Results:**

125 of the 230 participants who completed the survey had diverse levels of experience in IIF pattern recognition on HEp-2 cells ranging from <1 year to >10 years of experience (average >10 years). Participants had a high concordance in correctly classifying the classical ANA IIF patterns: ranging from 95.2 % for centromere to 74.4 % for nucleolar patterns. The unmixed DFS pattern was recognized with significantly lower accuracy (~50 %; *p* < 0.05). However, less than 10 % correctly identified mixed patterns derived from the sera containing both clinically relevant ANA and anti-DFS70 antibodies.

**Conclusions:**

Recognizing the DFS ANA IIF pattern and mixed IIF patterns composed of DFS + clinically relevant ANA patterns poses a significant challenge. Consequently, it seems imperative that DFS-specific immunoassays should be used to confirm the presence of anti-DFS70 antibodies before definitive results are reported to physicians.

## Introduction

The presence of anti-cellular antibodies [1], commonly referred to as antinuclear antibodies (ANA), directed against intracellular antigens is a hallmark of ANA-associated rheumatic diseases (AARD) [2]. ANA are most commonly detected by the indirect immunofluorescence (IIF) assay on HEp-2 cell substrates [3]. However, not all ANA are associated with AARD thus complicating the interpretation and use of the test results [4]. Anti-dense fine speckled 70 (anti-DFS70) antibodies were initially identified as generating a specific ANA IIF pattern from a patient with interstitial cystitis [5], but were later associated with various other conditions (reviewed in [6]). The DFS pattern as detected by IIF on HEp-2 cells has been associated with several inflammatory diseases but is most commonly observed in individuals that do not have an AARD and even in apparently healthy individuals (HI). Consequently, the accurate identification and correct reporting of this IIF pattern is of utmost importance. This pattern has been recognized by several international study groups for the detection of ANA [1, 7, 8] and the DFS IIF pattern has recently been assigned the AC-02 nomenclature and designated as a competency level recognition pattern by the International Consensus on ANA Pattern (ICAP, http://www.anapatterns.org/) Committee.

With respect to the prognostic and long-term outcome of individuals with anti-DFS70 antibodies, it was reported that none of 40 HI with isolated anti-DFS70 reactivity developed an AARD within an average 4-year follow-up [9]. Therefore, it was suggested that the presence of isolated anti-DFS70 antibodies could be used to help to rule out a diagnosis of AARD including systemic lupus erythematosus (SLE), systemic sclerosis (SSc), inflammatory idiopathic myopathies (IIM), Sjögren’s syndrome (SjS) and mixed connective tissue disease (MCTD) [9–12]. In previous studies, it was found that anti-DFS70 antibodies are more prevalent in females than in males, a finding that is important since females are also predominately affected by AARD [10].

Since ANA and related autoantibodies are generally considered useful biomarkers for AARD (which have low prevalences) and are included in the classification criteria for SLE [13], MCTD [14], SjS [15] and SSc [16], ANA testing on HEp-2 substrates outside a proper clinical framework may yield a sizable portion of ANA-positive individuals without consistent evidence of AARD. In this context, ANA testing may purportedly lead to inappropriate referrals to tertiary care specialists, as well as anxiety in patients and physicians alike [9] and, perhaps, inappropriate and potentially toxic therapies [17]. Therefore, the concept of utilizing anti-DFS70 antibodies as a diagnostic or prognostic discriminator of ANA-positive subjects with and without AARD is appealing, but reliable data from various clinical and diagnostic laboratory sites are mandatory to support the clinical use of this marker. Since proper reading of the DFS pattern, is crucial to ensure its usefulness in supporting clinical diagnosis, the objective of this study was to use an internet-based survey to assess how accurately the DFS IIF pattern was recognized by experienced technologists.

## Materials and methods

### Creation of samples

Two serum samples with anti-DFS70 antibodies and four samples with typical AARD-associated antibodies from AARD patients were used to produce a serum panel for IIF studies and the web-based survey. The two anti-DFS70-positive samples (confirmed positive by QUANTA Flash DFS70 chemiluminescence immunoassay) were monospecific and showed no additional ANA reactivity. The four samples with AARD-associated antibodies exhibited established clinically relevant IIF patterns (centromere, nucleolar, speckled, homogeneous) and produced strong intensity fluorescence at 1:80 dilution. To obtain mixed patterns, these four samples were mixed with one of the anti-DFS70 positive samples in different ratios to determine the potential masking effect of anti-DFS70 antibodies on other patterns (Table 1).

**Table 1:** Mixed pattern experiment design for blending samples

Established sample	Sample 1: mix ratio (sample/DFS70)	Sample 2: mix ratio (sample/DFS70)	Sample 3: mix ratio (sample/DFS70)	Sample 4: mix ratio (sample/DFS70)	Sample 5: mix ratio (sample/DFS70)
Centromere	C1 (10/90)	C2 (25/75)	C3 (50/50)	C4 (75/25)	C5 (90/10)
Speckled	S1 (10/90)	S2 (25/75)	S3 (50/50)	S4 (75/25)	S5 (90/10)
Homogeneous	H1 (10/90)	H2 (25/75)	H3 (50/50)	H4 (75/25)	H5 (90/10)
Nucleolar	N1 (10/90)	N2 (25/75)	N3 (50/50)	N4 (75/25)	N5 (90/10)

Each established pattern sample (centromere, nucleolar, speckled, homogeneous) was blended with a known anti-DFS70-positive sample in different ratios

### Immunofluorescence assays (IIF) and survey

High-resolution color digital IIF images (300 DPI) were captured using the automated IIF NOVA View® instrument (Inova Diagnostics, San Diego, CA). Ten images were posted in an anonymous, international, internet-based interpretive survey (https://www.surveygizmo.com/) as completed by IIF technologists. Four of the images in the survey were from serum samples with classic ANA IIF, two of the images were from samples with a DFS IIF ANA pattern and monospecific anti-DFS70 antibodies, and four images were from sera with mixed patterns referred to above. The survey included multiple choice selections: homogeneous, DFS, centromere, nucleolar, speckled, other, or unrecognizable. Incomplete survey results were excluded from the final result analysis.

### Chemiluminescence anti-DFS70 antibody assay

The QUANTA Flash^®^ DFS70 (Inova Diagnostics, San Diego, USA) is a novel chemiluminescence assay (CIA) that uses recombinant DFS70 (expressed in *E. coli*) bound to paramagnetic beads and is designed for the BIO-FLASH^®^ instrument (Biokit s.a., Barcelona, Spain) [18]. The principles and protocols of the assay system have been previously described [19, 20]. In brief, the relative light units (RLUs) measured are proportional to the amount of isoluminol conjugate that is bound to the human IgG, which in turn is proportional to the amount of anti-DFS70 antibodies bound to the antigen on the beads. Samples above the analytical measuring range were diluted to determine the exact concentration of anti-DFS70 antibodies.

### Immunoadsorption of anti-DFS70 antibodies

Anti-DFS70 antibodies were blocked using NOVA Lite HEp-2 Select® (Inova Diagnostics) which uses recombinant DFS70 antigen in the dilution buffer to prevent anti-DFS70 antibodies from binding the target antigen on the HEp-2 cell substrate. Prior to application of the diluted samples onto the HEp-2 substrate, diluted samples were incubated for 30 min. The subsequent assay procedure was identical to conventional IIF procedures. Results were interpreted using NOVA View (Inova Diagnostics), an automated digital image analysis system which is used for acquiring, analyzing, and interpreting ANA testing on HEp-2 cells, based on measured light intensity units (LIU) and pattern recognition.

### Statistical analysis

Data were statistically evaluated using the Analyse-it software (Version 2.03; Analyse-it Software, Ltd., Leeds, UK). Mann–Whitney *U* test and Fisher exact test were carried out to analyze the difference between groups. For all statistical tests, *p* values <0.05 were considered as significant.

## Results

**Fig. 1:**
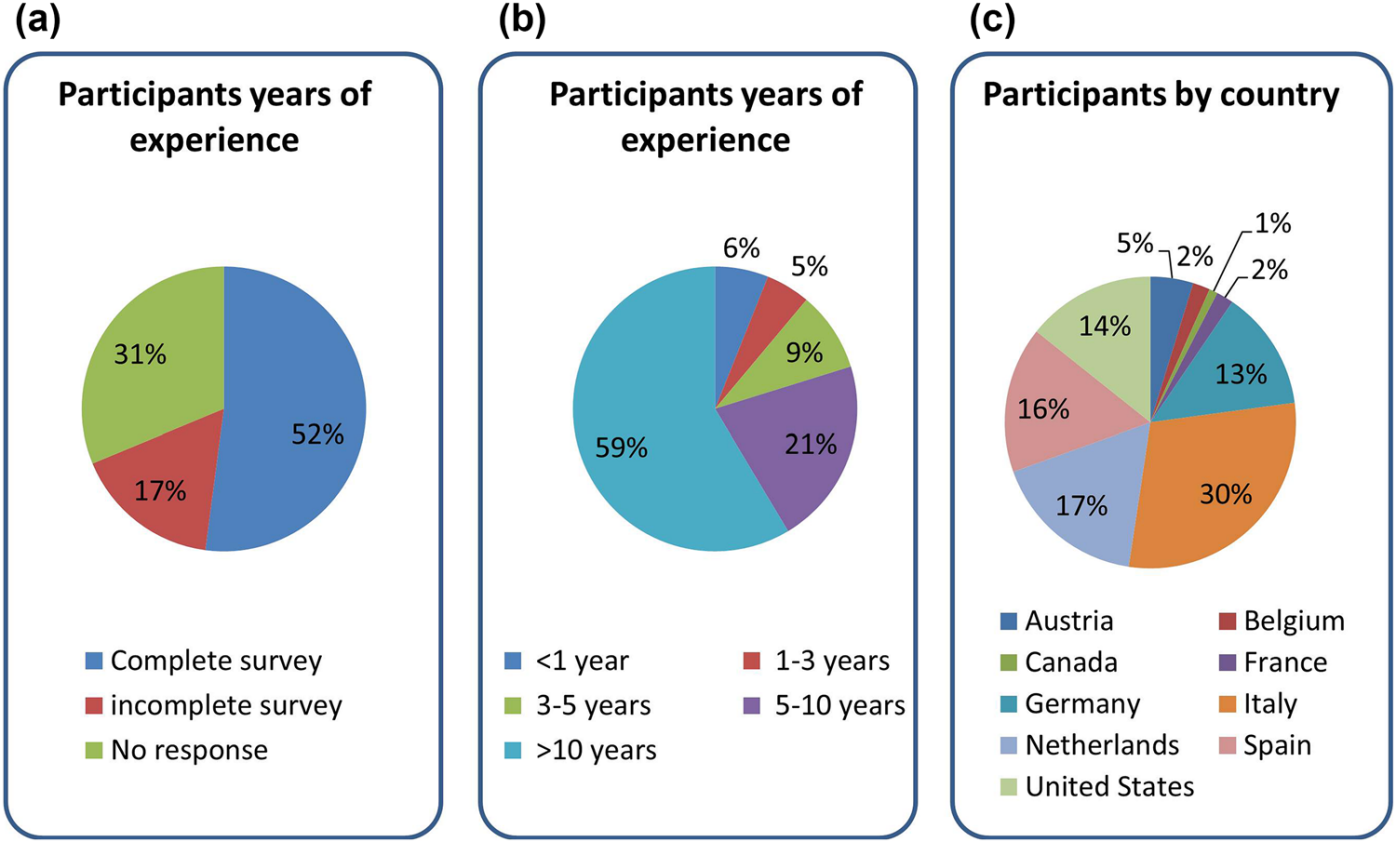
Summary of survey response. **a** The survey response rate is shown indicating that most of the invited participants completed the survey. **b** The distribution of the experience of all participants exhibits a long experience of most participants. **c** The majority of participants were from Italy, followed by Netherlands and Spain

**Fig. 2:**
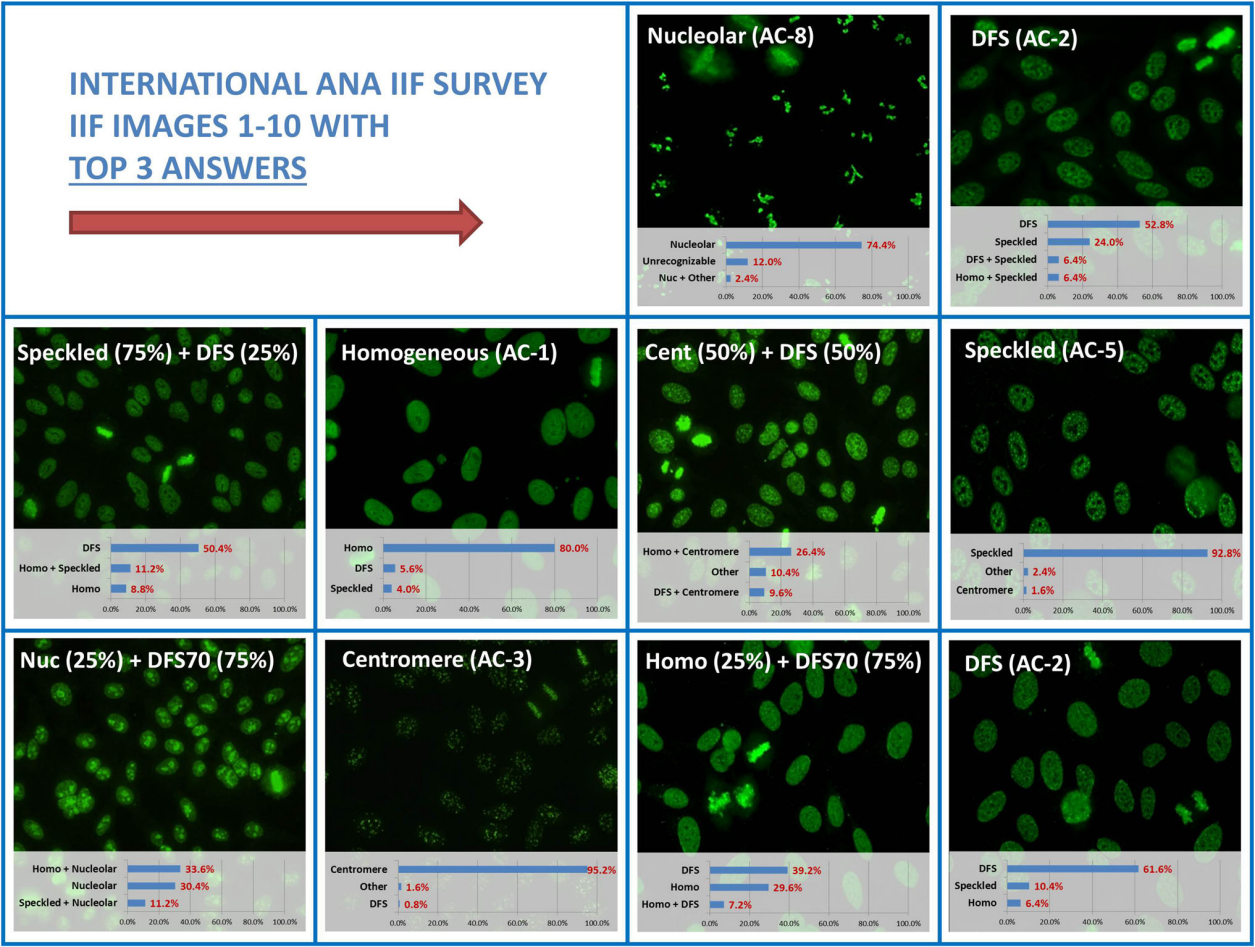
Results of the ten indirect immunofluorescence (IIF) images used in the survey. The ten patterns which were used and the results obtained from the survey are shown. Most notably, the major challenge was found with the mixed patterns. Patterns are indicated according to the recent nomenclature of the International Consensus on ANA Pattern (ICAP)

**Fig. 3:**
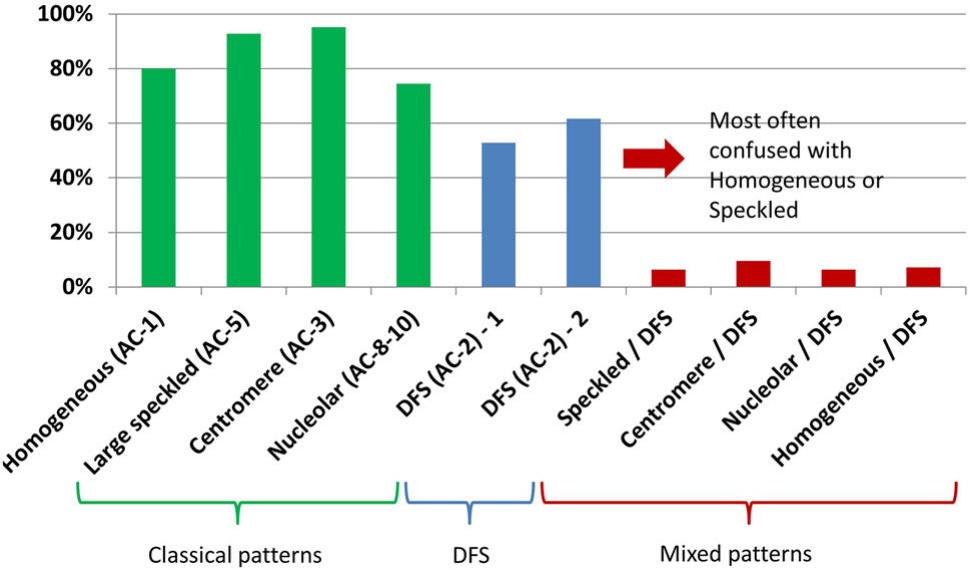
Summary of pattern recognition results. The four classical patterns: homogeneous, large speckled, centromere and nucleolar were recognized with high accuracy. The two samples with the dense fine speckled (DFS) pattern were recognized with significantly lower accuracy. However, the major challenge was found with the mixed patterns. Patterns are indicated according to the recent nomenclature of the International Consensus on ANA Pattern (ICAP)

125 of the 230 participants from several countries who completed the survey had diverse levels of experience in IIF pattern recognition on HEp-2 cells ranging from <1 year to >10 years (average >10 years). Most participants had more than 10 years of experience (details are summarized in Fig. 1). Participants had a high concordance in correctly classifying the classical ANA IIF patterns: ranging from 95.2 % for centromere to 74.4 % for nucleolar patterns. The unmixed DFS pattern was recognized with significantly lower accuracy (~50 %; *p* < 0.05). However, less than 10 % correctly identified mixed patterns derived from the sera containing both clinically relevant and anti-DFS70 antibodies (Figs. 2, 3).

When the immunoadsorption for DFS70 was used on samples with isolated anti-DFS70 antibodies, the DFS pattern was adsorbed and the IIF result was negative. On the mixed samples, anti-DFS70 antibodies were also blocked and the other clinically relevant pattern was revealed.

## Discussion

Although ANAs represent biomarkers with demonstrated high value in the diagnosis of AARD, not all ANAs are associated with AARD [4]. One such autoantibody, anti-DFS, was first described in 1994 and has been historically associated with various other diseases and even in apparently HI (reviewed in [21]). The detection of anti-DFS70 autoantibodies has primarily depended on detection of the typical DFS IIF staining pattern, and in some laboratories followed by immunoblot, immunoprecipitation and, more recently, analyte-specific immunoassays such as ELISA and chemiluminescence [18, 22, 23]. It has been reported that the frequency of anti-DFS70 antibodies in routine laboratories is similar to that of other important AARD autoantibodies such as anti-dsDNA antibodies [24–26]. As pointed out in our study and another report [21, 27], the detection of even isolated anti-DFS70 by IIF is likely not performed with high precision in diagnostic laboratories. This is complicated further by emerging evidence that anti-DFS70 do not always occur in isolation but may be seen in the context of other ANA which, as we have shown in this study, leads to a further significant decrease in IIF reading accuracy. Hence, the observations from our study indicate that detection of anti-DFS70 autoantibodies should not rely exclusively on the interpretation of the IIF staining pattern, but should be supported by analyte-specific immunoassays.

Although a distinctive clinical association is unreported, isolated anti-DFS70 antibodies have been proposed as a useful biomarker for the exclusion of AARD [10–12]. This suggestion has mainly been based on the observation that isolated anti-DFS antibodies are more prevalent in healthy individuals (HI) than in AARD patients, and that anti-DFS-positive HI did not develop AARD after clinical follow-up of 4 years [9]. Additional support for the hypothesis came from the observation that approximately 30 % of ANA-positive samples from HI have anti-DFS70 antibodies [9, 28] compared to 0 % in ANA-positive individuals with AARD. Anti-DFS70 antibodies have been reported in approximately 3 % of SLE patients [11], but are usually accompanied by other SLE-associated antibodies such as anti-dsDNA, anti-SS-A/Ro60, anti-RNP or anti-Sm [11, 18, 29]. However, excluding AARD in a patient with a positive ANA and protean signs and symptoms of an AARD requires careful consideration of the ANA specificity. Consequently, the identification of anti-DFS70 antibodies should be based on validated procedures.

Since a positive ANA test result is an important component in the diagnosis of patients with possible AARD, clinically irrelevant ANA-positive results, including those related to anti-DFS70 antibodies, have the potential to lead to an incorrect diagnosis, attending concern and anxiety in patients and physicians [9], prescriptions of inappropriate and potentially toxic therapeutics [30]. Hence, as our study shows, it is imperative that samples with DFS staining pattern identified by IIF should be tested for anti-DFS70 antibodies by a specific immunoassay (i.e., ELISA or CIA) and the result should be included in the laboratory report. In addition, it is advisable that clinicians should not over-interpret positive ANA results in patients with anti-DFS70 antibodies alone but should focus on whether anti-DFS70 is present in isolation (i.e., complimented by the detection of other disease-specific autoantibodies) and more importantly, on the presence or absence of clinical signs and symptoms of AARD.

The results of our survey need to be considered with caution based on the following shortcomings. First, reading patterns using a picture posted on the web might not deliver the same accuracy as using a microscope. Second, we only used one commercial HEp-2 cell substrate. Significant differences have been described between various IIF staining patterns on HEp-2 cells from different manufacturers [24, 27] and, therefore, it remains unclear if the DFS pattern can be recognized with similar accuracy using slides from a variety of manufacturers. Such variations might be attributed to the media and culture conditions, a variety of fixation methods used for manufacturing of the cell substrates, and various other technical aspects of slide preparation [27]. Although previous data [18] indicate that the DFS pattern can be identified on slides from a number of ANA kit manufacturers, more samples need to be analyzed and a more comprehensive multi-center study is needed to arrive at a conclusion, especially since conflicting results have been published [27].

## Conclusions

Recognizing the DFS ANA IIF pattern and mixed IIF patterns composed of DFS plus clinically relevant ANA poses a significant challenge. Consequently, it seems imperative that specific immunoassays are needed to confirm the presence of anti-DFS70 antibodies before definitive results are reported to clinicians.

## Abbreviations

AARD: ANA-associated rheumatic disease
ANA: Antinuclear antibody
DFS: Dense fine speckled
IIF: Indirect immunofluorescence
LEDGF: Lens-derived epithelium growth factor
HI: Healthy individuals
LR: Likelihood ratio
RA: Rheumatoid arthritis
SLE: Systemic lupus erythematosus
SSc: Systemic sclerosis

## References

[1] Agmon-Levin N, Damoiseaux J, Kallenberg C (2013). International recommendations for the assessment of autoantibodies to cellular antigens referred to as anti-nuclear antibodies. Ann Rheum Dis.

[2] Mahler M, Fritzler MJ (2010). Epitope specificity and significance in systemic autoimmune diseases. Ann N Y Acad Sci.

[3] Meroni PL, Schur PH (2010). ANA screening: an old test with new recommendations. Ann Rheum Dis.

[4] Slight-Webb S, Lu R, Ritterhouse LL (2016). Autoantibody-positive healthy individuals display unique immune profiles that may regulate autoimmunity. Arthritis Rheumatol.

[5] Ochs RL, Muro Y, Si Y (2000). Autoantibodies to DFS 70 kd/transcription coactivator p75 in atopic dermatitis and other conditions. J Allergy Clin Immunol.

[6] Ganapathy V, Casiano CA (2004). Autoimmunity to the nuclear autoantigen DFS70 (LEDGF): what exactly are the autoantibodies trying to tell us?. Arthritis Rheum.

[7] Chan EK, Damoiseaux J, Carballo OG (2015). Report of the first international consensus on standardized nomenclature of antinuclear antibody HEp-2 cell patterns 2014–2015. Front Immunol.

[8] Damoiseaux J, von Muhlen CA, Garcia-de la Torre I (2016). International consensus on ANA patterns (ICAP): the bumpy road towards a consensus on reporting ANA results. Autoimmun Highlights.

[9] Mariz HA, Sato EI, Barbosa SH (2011). Ana HEp-2 pattern is a critical parameter for discriminating ana-positive healthy individuals and patients with autoimmune rheumatic diseases. Arthritis Rheum.

[10] Watanabe A, Kodera M, Sugiura K (2004). Anti-DFS70 antibodies in 597 healthy hospital workers. Arthritis Rheum.

[11] Muro Y, Sugiura K, Morita Y, Tomita Y (2008). High concomitance of disease marker autoantibodies in anti-DFS70/LEDGF autoantibody-positive patients with autoimmune rheumatic disease. Lupus.

[12] Mahler M, Hanly JG, Fritzler MJ (2011). Importance of the dense fine speckled pattern on HEp-2 cells and anti-DFS70 antibodies for the diagnosis of systemic autoimmune diseases. Autoimmun Rev.

[13] Tan EM, Cohen AS, Fries JF (1982). The 1982 revised criteria for the classification of systemic lupus erythematosus. Arthritis Rheum.

[14] Cappelli S, Bellando RS, Martinović D (2012). “To be or not to be,” ten years after: evidence for mixed connective tissue disease as a distinct entity. Semin Arthritis Rheum.

[15] Vitali C, Bombardieri S, Jonsson R (2002). Classification criteria for Sjogren’s syndrome: a revised version of the European criteria proposed by the American-European Consensus Group. Ann Rheum Dis.

[16] Johnson SR, Fransen J, Khanna D (2012). Validation of potential classification criteria for systemic sclerosis. Arthritis Care Res (Hoboken).

[17] Fritzler MJ (2016). Choosing wisely: review and commentary on anti-nuclear antibody (ANA) testing. Autoimmun Rev.

[18] Mahler M, Parker T, Peebles CL (2012). Anti-DFS70/LEDGF antibodies are more prevalent in healthy individuals compared to patients with systemic autoimmune rheumatic diseases. J Rheumatol.

[19] Mahler M, Radice A, Sinico RA (2011). Performance evaluation of a novel chemiluminescence assay for detection of anti-GBM antibodies: an international multicenter study. Nephrol Dial Transplant.

[20] Mahler M, Radice A, Yang W (2012). Development and performance evaluation of novel chemiluminescence assays for detection of anti-PR3 and anti-MPO antibodies. Clin Chim Acta.

[21] Mahler M, Meroni PL, Andrade LE (2016). Towards a better understanding of the clinical association of anti-DFS70 autoantibodies. Autoimmun Rev.

[22] Bizzaro N, Tonutti E, Tampoia M (2015). Specific chemoluminescence and immunoadsorption tests for anti-DFS70 antibodies avoid false positive results by indirect immunofluorescence. Clin Chim Acta.

[23] Miyara M, Albesa R, Charuel JL (2013). Clinical phenotypes of patients with anti-DFS70/LEDGF antibodies in a routine ANA referral cohort. Clin Dev Immunol.

[24] Mahler M, Ngo JT, Schulte-Pelkum J (2008). Limited reliability of the indirect immunofluorescence technique for the detection of anti-Rib-P antibodies. Arthritis Res Ther.

[25] Mahler M, Silverman ED, Fritzler MJ (2010). Novel diagnostic and clinical aspects of anti-PCNA antibodies detected by novel detection methods. Lupus.

[26] Mariz HA, Sato EI, Barbosa SH (2011). Pattern on the antinuclear antibody-HEp-2 test is a critical parameter for discriminating antinuclear antibody-positive healthy individuals and patients with autoimmune rheumatic diseases. Arthritis Rheum.

[27] Bizzaro N, Tonutti E, Villalta D (2011). Recognizing the dense fine speckled/lens epithelium-derived growth factor/p75 pattern on HEP-2 cells: not an easy task! Comment on the article by Mariz et al. Arthritis Rheum.

[28] Ogawa Y, Sugiura K, Watanabe A (2004). Autoantigenicity of DFS70 is restricted to the conformational epitope of C-terminal alpha-helical domain. J Autoimmun.

[29] Schmeling H, Mahler M, Levy DM (2015). Autoantibodies to dense fine speckles in pediatric diseases and controls. J Rheumatol.

[30] Narain S, Richards HB, Satoh M (2004). Diagnostic accuracy for lupus and other systemic autoimmune diseases in the community setting. Arch Intern Med.

